# GC–MS Combined with Fast GC E-Nose for the Analysis of Volatile Components of Chamomile (*Matricaria chamomilla* L.)

**DOI:** 10.3390/foods13121865

**Published:** 2024-06-13

**Authors:** Jiayu Lu, Zheng Jiang, Jingjie Dang, Dishuai Li, Daixin Yu, Cheng Qu, Qinan Wu

**Affiliations:** 1 Jiangsu Collaborative Innovation Center of Chinese Medicinal Resources Industrialization, Nanjing University of Chinese Medicine, Nanjing 210023, China; lujiayu5210@163.com (J.L.); jyjiangzheng@163.com (Z.J.); 20223110@njucm.edu.cn (J.D.); 20210651@njucm.edu.cn (D.L.); yudaixin0616@163.com (D.Y.); 2 State Key Laboratory on Technologies for Chinese Medicine Pharmaceutical Process Control and Intelligent Manufacture, Nanjing University of Chinese Medicine, Nanjing 210023, China; 3 School of Pharmacy, Nanjing University of Chinese Medicine, Nanjing 210023, China

**Keywords:** *Matricaria chamomilla*, HERACLES Neo, GC-MS, flavor, volatile components

## Abstract

Chamomile has become one of the world’s most popular herbal teas due to its unique properties. Chamomile is widely used in dietary supplements, cosmetics, and herbal products. This study aimed to investigate the volatile aromatic components in chamomile. Two analytical techniques, gas chromatography–mass spectrometry (GC-MS) and an ultra-fast gas chromatography electronic nose, were employed to examine samples from Xinjiang (XJ), Shandong (SD), and Hebei (HB) in China, and imported samples from Germany (GER). The results revealed that all chamomile samples contained specific sesquiterpene compounds, including α-bisabolol, bisabolol oxide, bisabolone oxide, and chamazulene. Additionally, forty potential aroma components were identified by the electronic nose. The primary odor components of chamomile were characterized by fruity and spicy notes. The primary differences in the components of chamomile oil were identified as (*E*)-*β*-farnesene, chamazulene, *α*-bisabolol oxide B, spathulenol and *α*-bisabolone oxide A. Significant differences in aroma compounds included geosmin, butanoic acid, 2-butene, norfuraneol, *γ*-terpinene. This study demonstrates that GC–MS and the ultra-fast gas chromatography electronic nose can preliminarily distinguish chamomile from different areas, providing a method and guidance for the selection of origin and sensory evaluation of chamomile. The current study is limited by the sample size and it provides preliminary conclusions. Future studies with a larger sample size are warranted to further improve these findings.

## 1. Introduction

Chamomile (*Matricaria chamomilla* L.) is one of the most important, widely used aromatic and medicinal plants, belonging to the Asteraceae family. It has been used both as food and medicine for its aroma, anti-inflammatory, analgesic, and antibacterial properties [1,2]. Its extracts, oils, and teas are utilized worldwide to treat various ailments, including stomach issues, spasms, dermatitis, chronic headaches, constipation, anhidrosis, joint swelling, and urinary system diseases [3,4]. Chamomile is also used in skincare and cosmetic products, such as face creams, shampoos, and conditioners. Additionally, it is employed in traditional medicines of Asian countries including India, China, and Japan. In Chinese traditional medicine, it is incorporated into proprietary medicines [5]. Chamomile is widely cultivated, primarily in Eastern Europe, North America, South America, and parts of Asia. Market assessments forecast that global demand for chamomile will exceed USD 400 billion by 2025 [6].

The most commonly used components of chamomile are its essential oils, which promote relaxation and provide calming effects on the nervous system. Additionally, its aromatic components are extracted for flavoring beverages and as additives in perfumes [7,8]. Chamomile’s volatile organic compounds (VOCs), mainly consisting of monoterpenes and sesquiterpenes, include bisabolol and its oxides, chamazulene, farnesene, and cineole. These components are primarily found in the volatile oil and have calming, antidepressant, and antimicrobial effects, underscoring their substantial market value and research significance [9]. Research on chamomile’s volatile components typically utilizes gas chromatography–mass spectrometry (GC-MS), requiring extraction of volatile oils and involving tedious sample preparation and time-consuming analysis [10]. However, this method lacks the capability to accurately gauge or describe the aroma [11]. Volatile components influence the quality of floral teas, with aromatic teas generally scoring higher in sensory evaluations [12,13]. In addition to traditional olfactory sensory evaluations, ultra-fast gas chromatography electronic noses (E-noses) have been used in the sensory evaluation of food and pharmaceuticals [14]. These devices can identify unknown odor types and qualitatively analyze volatile components within 1–3 min. Research on dry ginger’s odor components revealed that its overall aroma is spicy and fragrant, aligning with its sensory evaluation [15]. Additionally, the high-temperature extraction of volatile oils often degrades thermosensitive metabolites through carbocation formation or concerted cyclic rearrangements into their structural analogs, altering the composition [16,17]. Therefore, developing a rapid, non-destructive method for analyzing and describing the aroma of chamomile is essential for assessing its overall aroma information and quality.

This study employed gas chromatography–mass spectrometry (GC-MS) and ultra-fast gas chromatography electronic nose technology to analyze the volatile oil components and aroma profiles of chamomile. This is the first study to use an electronic nose to analyze the aroma of chamomile. These results provide a comprehensive analysis of chamomile’s volatile components, contributing to a better understanding of its aroma compounds and sensory evaluations.

## 2. Materials and Methods

### 2.1. Instruments and Reagents

The HERACLES Neo ultra-fast gas chromatography electronic nose (Alpha Mos, Toulouse, France), equipped with a PAL RSI automatic headspace sampler, MXT-5 non-polar capillary column, and MXT-1701 medium-polarity capillary column, was used for the aroma analysis of chamomile. Additionally, the GA-380N low-noise air pump and the GH-380 high-purity hydrogen generator (Beijing Zhong Xing Hui Li Technology Development Co., Ltd., Beijing, China) were utilized for the analysis. The Agilent 7890B gas chromatograph and Agilent 7000C triple quadrupole mass spectrometer (Agilent Technologies, Santa Clara, CA, USA) were used for gas phase and mass spectrometric analysis of chamomile volatile oils.

An electronic analytical balance (AUX220, Shimadzu Corporation, Kyoto, Japan) was used for weighing. Sample drying was performed using an electric hot air oven (9140A DHG, Shanghai Jinghong Experimental Equipment Co., Ltd., Shanghai, China). A temperature-regulated electric heating mantle (PTHW3000 mL, Gong Yi Yu Hua Instruments Co., Ltd., Gongyi, China) was employed for the heating process. The SAGA-10TY laboratory ultrapure water system (Nanjing EasyPure Development Co., Ltd., Nanjing, China) provided ultrapure water for the experiments.

Mixed reference substances used in the experiment included n-alkanes (lot A0168401, containing 0.02–53.27 g of normal alkanes (C6–C16) per 100 g, Restek Corporation, Bellefonte, PA, USA), and a C7-C40 mixed standard (lot number S08HB193959, Shanghai Yuan Ye Co., Ltd., Shanghai, China). The n-hexane (lot K2212140) and n-hexadecane (lot J1217041) were sourced from Aladdin Chemical Reagents Co., Ltd., Nanjing, China, and the anhydrous sodium sulfate (lot 1503023670) was obtained from Nanjing Chemical Reagents Co., Ltd., Nanjing, China.

### 2.2. Chamomile Samples

**Figure 1:**
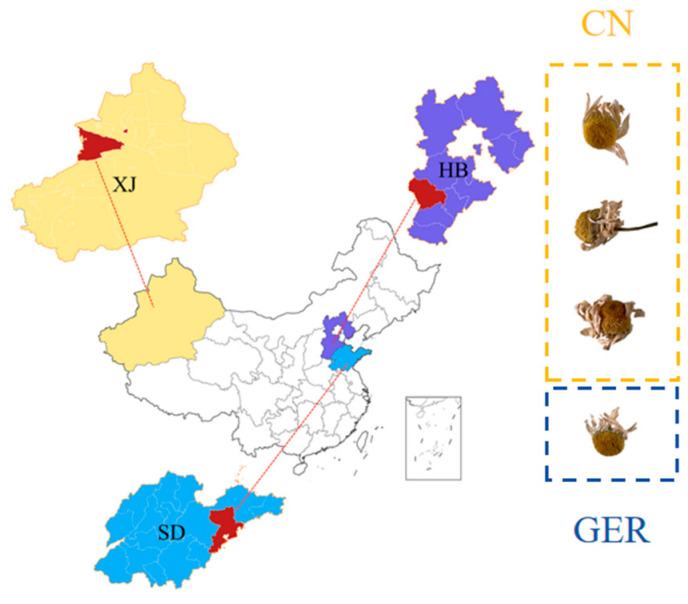
Regional information of chamomile from China (SD: Shandong; XJ: Xinjiang; HB: Hebei); CN: Samples from China; GER: Samples from Germany.

A total of 15 chamomile samples were collected in 2023 from major chamomile-producing areas in China. The samples originated from Xinjiang (XJ), Shandong (SD), and Hebei (HB), with geographical coordinates ranging from 80°09′ to 121°00′ East and 35°35′ to 49°10′ North (Figure 1). The German samples consisted of three different brands of chamomile collected in Germany. Each chamomile sample was ground into a fine powder using a high-speed grinder, and passed through a No. 1 sieve (850 µm ± 29 µm) as specified in the Chinese Pharmacopoeia for experimental use.

### 2.3. Determination of Volatile Oil Content

The method is based on the determination of volatile oil content as described in the Chinese Pharmacopoeia, with modifications [18]. Approximately 75 g of the sample (equivalent to 0.5 to 1.0 mL of volatile oil) were weighed and placed in a round-bottom flask. Then, 1500 mL of ultrapure water (or an appropriate amount) and a few zeolites were added. After shaking and mixing, the flask was connected to a volatile oil determiner and a reflux condenser. Water was added from the top of the condenser until it filled the scale part of the volatile oil determiner and overflowed into the flask. The mixture was then slowly heated to boiling with a temperature-regulated electric heating mantle and maintained at a consistent boil for 4 h [19]. The piston at the bottom of the determiner was opened after a short pause, and water was slowly released until the top of the oil layer reached 5 mm above the zero mark on the scale. The piston was opened again to lower the oil layer until its upper end was level with the zero mark, after the solution had been left to stand for 1 h. The volume of volatile oil was then read, and the content (%) of volatile oil in the test sample was calculated.

### 2.4. Volatile Oil GC–MS Analysis

55 μL of hexadecane was diluted to 50 mL with n-hexane to serve as the internal standard solution. Chamomile essential oil was dried over anhydrous sodium sulfate for the test sample solution. An amount of 10 μL of the essential oil was then extracted and diluted to 1 mL with the internal standard solution, serving as the sample solution for analysis.

The gas chromatographic conditions were based on the method described by Elahe Piri et al., with some modifications [20]: split mode injection with an injection volume of 1 μL, carrier gas helium (purity 99.999%) at a flow rate of 1.0 mL/min, injector temperature 250 °C, and split ratio 30:1. The oven temperature program started at 50 °C, held for 3 min, then increased at 5 °C/min to 250 °C, and held for 5 min. The total GC runtime was 48 min. The chromatographic column used was an Agilent 19091S-433I HP-5ms Inert, with temperature limits of 0 °C to 325 °C (maximum 340 °C): 30 m × 250 μm × 0.25 μm.

The mass spectrometry conditions were as follows: the ionization method was electron ionization (EI) with a quadrupole temperature of 150 °C, ion source temperature of 230 °C, mass scan range *m*/*z* 30.0 to 500.0 amu, interface temperature 280 °C, and full scan mode at an electron energy of 70 eV.

### 2.5. Ultra-Fast Gas Chromatography Electronic Nose Analysis

#### 2.5.1. Single-Factor Investigation

Sample Quantity: The injection volume was set at 3000 μL, with an incubation temperature of 60 °C and an incubation time of 20 min. The impact of different quantities (0.2 g, 0.3 g, 0.4 g, 0.5 g, 1.0 g) of the same chamomile sample (ID S-7) on the chromatographic peak area was investigated. Results indicated that the peak area increased with the sample quantity. No noticeable increase in peak area was observed for sample quantities of 0.5 g or 1 g. Therefore, a sample weight of 0.5 g was selected based on the relationship between sample weight and peak area.

Injection Volume: A 0.5 g sample of chamomile was analyzed, with the incubation temperature set at 60 °C and the incubation time at 20 min. The effect of different injection volumes (1000 μL, 2000 μL, 3000 μL, 4000 μL, 5000 μL) on the odor chromatographic peak area was examined. Results showed that peak area increased with the injection volume, thus the chosen injection volume was 5000 μL.

Incubation Temperature: A 0.5 g sample of chamomile was analyzed, with an incubation time of 20 min and an injection volume of 5000 μL. The impact of various incubation temperatures (40 °C, 50 °C, 60 °C, 70 °C) on the odor chromatographic peak area was explored. As the temperature increased, the chromatographic peak response gradually increased. Chamomile contains aromatic volatile oils, and lower temperatures are closer to real environmental conditions. According to the instrument’s instructions, temperatures below 50 °C are considered low. The maximum chromatographic peak response was observed at 50 °C, so the incubation temperature was set at 50 °C.

Incubation Time: A 0.5 g sample of chamomile was analyzed, with an incubation temperature of 50 °C and an injection volume of 5000 μL. The influence of different incubation times (10 min, 15 min, 20 min, 25 min, 30 min) on the odor chromatographic peak area was assessed. The results demonstrated that the peak area became saturated and stable at an incubation time of 20 min. Therefore, the chosen incubation time was 20 min.

#### 2.5.2. Sample Analysis

A 0.5 g sample of chamomile was placed in a 20 mL headspace vial specifically designed for electronic nose analysis and sealed with a PTFE gasket. The HERACLES Neo Analyzer is an advanced, fast gas-phase electronic nose with a built-in pre-concentration trap [21], unlike the standard metal oxide semiconductor (MOS) electronic noses, which rely on metal oxide sensors. Initially, each sample is placed in an incubator to concentrate the volatile components in the gas phase of the headspace vial until equilibrium is reached. The gaseous sample is then transferred to the pre-concentration trap, where the captured components are quickly released and separated in the MXT-5 and MXT-1701 columns, allowing for rapid separation. The concentrated odors are detected using two hydrogen flame ionization detectors (FIDs). Alpha Soft-17.0 software is used to record the signals, and each sample is tested three times in parallel.

The instrument conditions were modified based on preliminary research conducted in our laboratory. The chromatographic columns used are a low polarity MXT-5 (5% diphenyl/95% dimethyl polysiloxane, 10 m × 0.18 mm, 0.4 μm) and a medium polarity MXT-1701 (14% cyanopropylphenyl/86% dimethyl polysiloxane, 10 m × 0.18 mm, 0.4 μm) metal capillary columns. The headspace vial volume is 20 mL; sample quantity is 0.5 g; injection volume is 5000 μL; shaking temperature is 50 °C; shaking time is 20 min; injection speed is 125 μL/s; injection duration is 45 s; injection port temperature is 200 °C; initial temperature of the capture trap is 40 °C; hydrogen is used as the carrier gas at a flow rate of 1.0 mL/min, with a capture trap diversion rate of 10 mL/min; the capture duration is 50 s; final temperature of the capture trap is 240 °C; initial column temperature is 50 °C. The temperature program ramps from 1 °C/s to 80 °C, then 3 °C/s to 250 °C, holding for 21 s. Acquisition time is 110 s; FID gain is 12 [15].

#### 2.5.3. Methodological Investigation

Precision: Six chamomile samples (ID S-7) were precisely weighed, each at 0.5 g, and analyzed under the detection conditions described in Section 2.5.2. Ten common peaks were identified, with their chromatographic peak areas and retention times recorded. Results showed that the RSD values for the peak areas of the ten common peaks ranged from 2.60% to 7.12%, and were all below 10.00%; RSD values for their retention times ranged from 0.03% to 0.14%, and were all below 3.0%, indicating good instrument precision.

Reproducibility: Six parallel preparations of chamomile samples (ID S-7) were precisely weighed. The analysis was conducted according to the method described in Section 2.5.2. Ten common peaks were recorded, along with their chromatographic peak areas and retention times. Results showed that the RSD values for the peak areas of the ten common peaks ranged from 1.15% to 6.94%, and were all below 10.00%; RSD values for their retention times ranged from 0.02% to 0.06%, and were all below 3.0%, indicating good reproducibility of the experiment.

Stability: The same chamomile sample (ID S-7) was precisely weighed at 0.5 g at 0, 2, 4, 8, 12, 24 h and analyzed under the detection conditions described in Section 2.5.2. Ten common peaks were recorded, along with their chromatographic peak areas and retention times. Results showed that the RSD values for the peak areas of the ten common peaks ranged from 1.34% to 8.63%, and were all below 10.00%. RSD values for their retention times ranged from 0.03% to 0.15%, and were all below 3.0%, indicating good stability within 24 h.

### 2.6. Qualitative and Quantitative Analysis

GC–MS analysis was employed to analyze a solution of n-alkanes (C7-C40) under identical conditions to calculate the retention index (RI) values of various volatile compounds in chamomile essential oil. The NIST 14 library was used to identify unknown compounds by comparing the mass spectral information with published RI values. Typically, higher R-matching values in the NIST library are considered the first indicator for identifying unknown compounds. Additionally, a difference of no more than 30 between experimental RI values and reported RI values is regarded as an important criterion for identification [22]. To quantitatively analyze different components, n-hexadecane was added as an internal standard in each sample. The relative content of each component in the essential oil was determined by the ratio of the analyzed peak area to the internal standard.

The flavor components analyzed by the fast gas chromatography electronic nose (e-nose) were calibrated using mixed n-alkanes (C6 to C16 standards [23]) for calculating RI values, and potential compounds were identified through comparison with the AroChemBase database.

### 2.7. Chemometric Analysis

Chemometric analyses were conducted using SIMCA–P software (Version 14.1, Umetrics, Sweden) for principal component analysis (PCA), partial least squares discriminant analysis (PLS–DA), and orthogonal partial least squares discriminant analysis (OPLS–DA). Discriminant factor analysis (DFA) was carried out using the HERACLES fast gas chromatography electronic nose software Alpha Soft (version 17.0, Toulouse, France).

## 3. Results

### 3.1. Chamomile Volatile Oil

The yield of essential oil from chamomile has always been a concern in agricultural science and the commodity economy, as higher content of volatile oils in chamomile is generally considered indicative of better quality [24].The essential oil content of 18 batches of chamomile herbs ranged from 0.43% to 1.38%, with an average of 0.80%. The average essential oil contents of chamomile samples from different areas were 0.87% for Xinjiang, 1.02% for Shandong, 0.52% for Hebei, and 0.87% for Germany.

### 3.2. GC–MS Identification of Chamomile Volatile Oil Components

In this experiment, 16 volatile components were identified from chamomile samples from different areas, including 13 terpenoids, one aliphatic hydrocarbon, one ester, and one ketone. The relative content of each substance was determined by comparing the peak area of each batch’s chromatographic peaks with that of the internal standard, as shown in Table S2. All the essential oils were blue, due to the formation of chamazulene under high-temperature conditions. During distillation, high temperatures can lead to the formation of chamazulene, which imparts a blue color to the oil. However, supercritical CO2 extraction avoids this type of thermal degradation. As a result, oils extracted using this method do not exhibit the blue coloration associated with chamazulene formation [25]. Among the volatile oil components of chamomile, besides chamazulene, the oxygenated sesquiterpenes are the most characteristic, with bisabolol oxide B, *α*-bisabolol, bisabolone oxide, and bisabolol oxide A being the most notable compounds [18]. Chamazulene offers photoprotective effects on the human keratinocyte cell line (HaCaT) and provides ultraviolet blocking capabilities [26]; α-Bisabolol inhibited glioblastoma cell migration and invasion by downregulating central mucoepidermoid tumor (c-Met); *α*-Bisabolol oxide A from chamomile flowers is reported to inhibit the migration of Caco-2 colon cancer cells and deactivate the vascular epidermal growth factor receptor-2 (VEGFR2) angiogenic enzymes [27].

**Figure 2:**
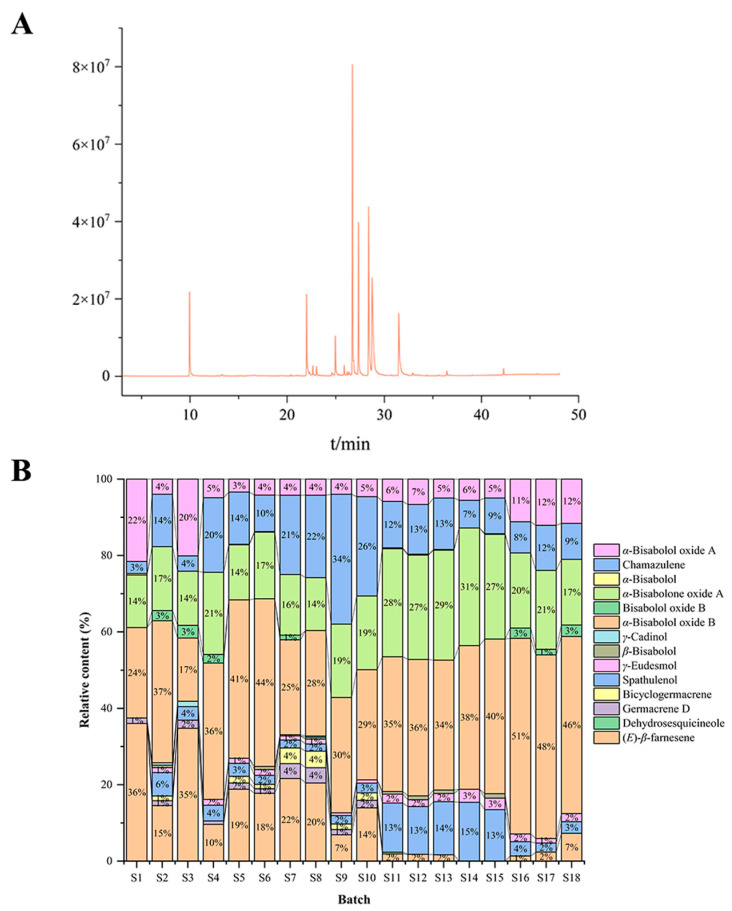
(**A**) Chromatogram example; (**B**) Histogram of volatile oil component distribution; S1–S18: Corresponding sample name from Table 1.

This experiment determined the average relative content of chamazulene from the three sources to be: XJ 0.71 μg/mL, SD 2.14 μg/mL, HB 0.45 μg/mL, and GER 0.22 μg/mL. The combined average relative content of bisabolol and its oxides (*β*-bisabolol, *α*-bisabolol oxide B, bisabolol oxide B, *α*-bisabolone oxide A, *α*-bisabolol, *α*-bisabolol oxide A) was: XJ 3.41 μg/mL, SD 4.68 μg/mL, HB 2.84 μg/mL, and GER 1.83 μg/mL. The results indicate that samples from Shandong (CN) have the highest oil content and levels of characteristic components, although these differences could be attributed to climate, soil, cultivation methods, etc. [28]. This can to some extent assess the quality of chamomile [29]. We generated a histogram to show intuitive representation of the differences in volatile oil components from different sources (Figure 2).

### 3.3. Discrimination of Chamomile by GC–MS

#### 3.3.1. PCA (Principal Component Analysis)

**Figure 3:**
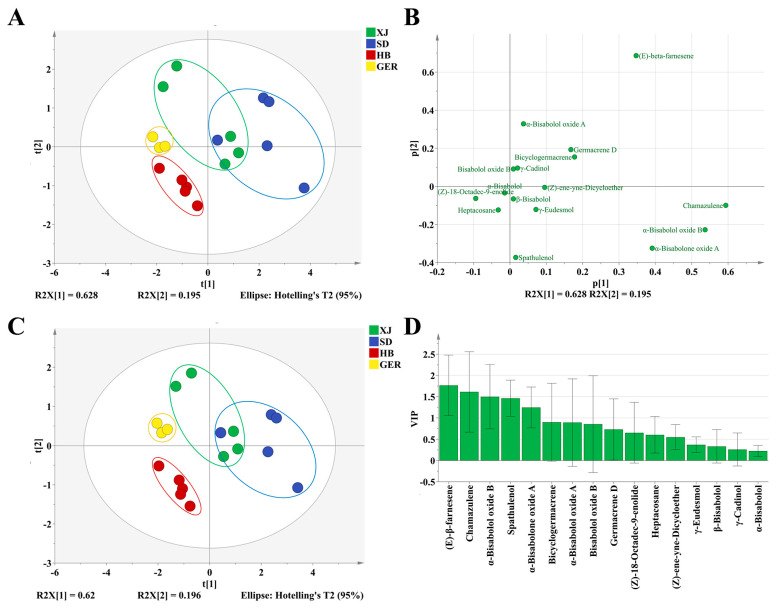
(**A**) Score plots of PCA Model; (**B**) Loading scatter plots of PCA Model; (**C**) Score plots of PLS–DA Model; (**D**) Variable importance factor (VIP) plots.

Principal component analysis (PCA) was conducted to investigate the differences in volatile oils of different chamomile samples. The common peak areas of chamomile volatile oils were used as variables, and PCA was performed using SIMCA 14.1 software, as shown in Figure 3A. The results revealed that the first two principal components accounted for 82.30% of the total variance (PC1 62.80%, PC2 19.50%), with R2X = 0.995 and a model predictive ability Q2(cum) = 0.746 (>0.5), indicating that the model can adequately reflect the sample information and explain and predict the total variance well.

The PCA was conducted using the first two principal components, t [1] and t [2], which together explain a significant portion of the variance in the dataset (R2X [1] = 0.628 and R2X [2] = 0.195). XJ samples are represented by green dots, forming a distinct cluster. This indicates a unique volatile oil profile that separates XJ samples from others. SD samples are represented by blue dots, forming a distinct cluster in the upper right quadrant. The separation from XJ samples suggests significant differences in the volatile oil components. HB samples are represented by red dots, and these samples form a separate cluster in the lower left quadrant. This indicates that HB samples also have a unique volatile oil profile. GER samples are represented by yellow dots, clustering tightly in the lower left region, suggesting that German chamomile samples have a distinct volatile profile that is closer to that of HB samples but still unique.

There is clear separation between the samples from different areas, indicating that the volatile oil profiles may be region-specific. Some overlap between XJ and SD samples suggests that, while they have distinct profiles, there might be some similarities in certain components. This differentiation can be attributed to various factors such as climate, soil, and cultivation practices. The clustering pattern highlights the potential for using PCA and volatile oil profiles to distinguish and authenticate chamomile samples from different geographical origins.

The scatter plot illustrates the loadings of volatile oil components from chamomile samples on the first two principal components (Figure 3). This analysis helps identify which specific volatile compounds are most influential in differentiating the chamomile samples. (*E*)-*β*-farnesene is positioned with high positive loadings on both p [1] and p [2], indicating it is a major contributor to the variance and differentiation of samples. Chamazulene and *α*-bisabol oxide B are positioned with high positive loadings on p [1], suggesting these compounds significantly influence the first principal component. *α*-bisabolol oxide A is positioned with high positive loadings on p [2], indicating its significant influence on the second principal component.

The loading scatter plots of the PCA effectively highlight the volatile compounds that may play vital roles in differentiating chamomile samples. The identified key compounds, such as (*E*)-*β*-farnesene, chamazulene, and *α*-bisabol oxide B, can be further investigated for their potential as markers for geographic origin.

#### 3.3.2. PLS–DA (Partial Least Squares Discriminant Analysis)

PCA is an unsupervised analysis method that cannot ignore intra-group differences and random errors when determining differential components. This study employed a supervised PLS–DA analysis model to explore the differences in volatile oils of chamomile from different areas and to identify potential differential compounds, as shown in Figure 3.

The results demonstrate that the 18 batches of chamomile were categorized into four groups, with samples from the same area clustering together. The model exhibited good explanatory and predictive capabilities, with R2X = 0.914, R2Y = 0.802, and Q2 = 0.566. The PLS–DA results provided preliminary validation of the PCA model. The 200 permutation tests yielded R^2^X = 0.192 and Q2Y = −0.548, both of which are below the thresholds of 0.3 and 0.05. This indicates that the model is reliable and not overfitted, as shown in Figure S1.

The VIP value is used to assess the relative importance of each variable in the PLS–DA model. A higher VIP value indicates a greater contribution of that variable to the model’s explanation. Variables with VIP values greater than 1 are not only statistically significant but also practically important. They may represent key biomarkers, environmental factors, or other important explanatory variables [21]. The analysis of the variable importance in projection (VIP) scores revealed that five components had VIP values greater than 1: (*E*)-β-farnesene, chamazulene, *α*-bisabolol oxide B, spathulenol, and *α*-bisabolone oxide A. These compounds are the most characteristic oxygenated sesquiterpenes and bicyclic terpenes in chamomile volatile oils, typically extracted from natural chamomile sources [18,28]. Therefore, these five components are considered potential differentiating substances for the volatile oils from the three distinct areas of chamomile (Figure 3).

### 3.4. Identification of Chamomile Using the Rapid Gas Chromatography Electronic Nose Method

#### 3.4.1. Rapid Gas Chromatography Electronic Nose Determination Results

**Figure 4:**
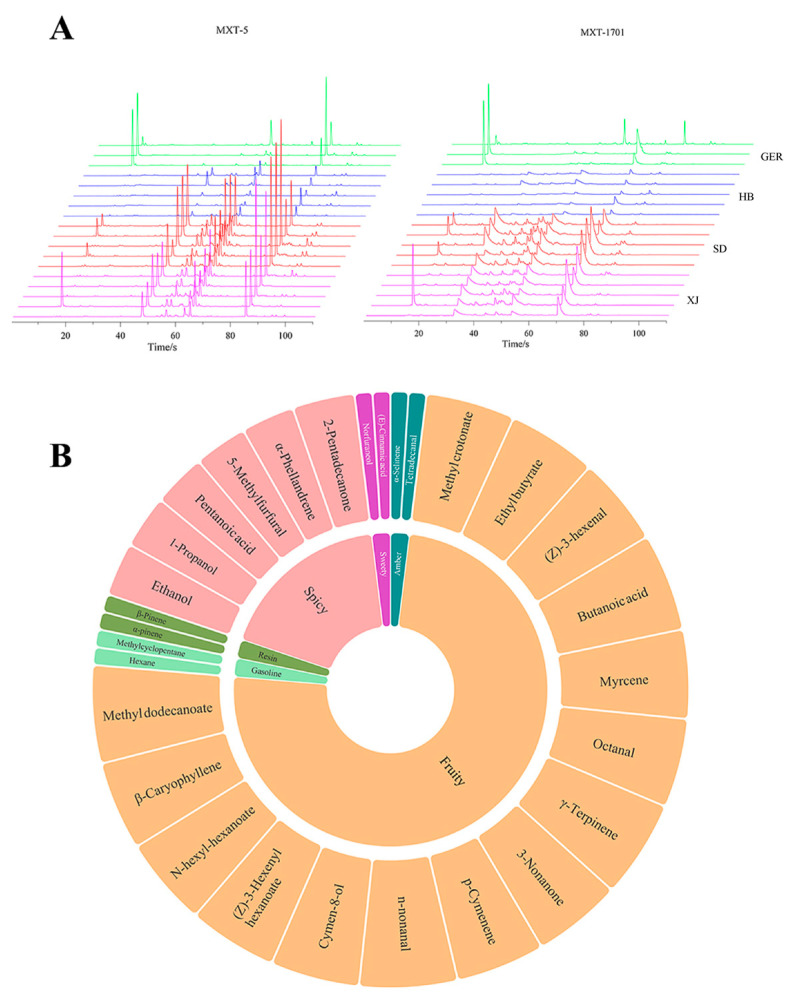
(**A**) Chamomile electronic nose fingerprint diagram; (**B**) Flavor wheel diagram of chamomile odor; inner circle: odor descriptors, outer circle: the identified compounds.

The retention index (RI) of the compounds obtained by GC–MS primarily range between 1500 and 2200, failing to capture data for compounds with retention indices below 1500. The ultra-fast gas chromatography electronic nose acts as a rapid olfactory analyzer that can enrich volatile components at low temperatures (50 °C) and collect information for compounds with RIs less than 1500. This system simulates human olfaction to provide sensory information about volatile components. Two sets of electronic nose chromatogram data were obtained and imported into Origin software (Version 2024), resulting in the generation of a chamomile medicinal material odor fingerprint map, as seen in Figure 4. It is observable that the main peak levels of the SD samples are higher than those from the other sources, indicating a more intense odor and greater aromaticity, suggesting a relatively higher quality. The chromatogram results indicate that chamomile odor components were well separated in the MXT-5 chromatographic column, which displayed a rich variety of components. Consequently, the MXT-5 column was selected as the primary identification column, with the MXT-1701 column as a secondary identification column.

Peaks with an area greater than 1000 and good separation were chosen for further analysis [15]. Forty odor compounds were identified within 110 s by Alpha Soft electronic nose software (Version 17.0) and the AroChemBase database (17.0), and their sensory characteristics were acquired. Figure 4 presents relevant information on these odor components, including nine terpenes, five esters, five aldehydes, three ketones, two alcohols, two alkanes, and three carboxylic acids. The results indicate that the aroma of chamomile is categorized into several major groups, with the primary aromas being fruity and spicy. Fifteen components were identified with a fruity odor, including methyl crotonate, ethyl butyrate, (*Z*)-3-hexenal, butanoic acid, myrcene, octanal, *γ*-terpinene, 3-nonanone, p-cymenene, n-nonanal, cymen-8-ol, (*Z*)-3-hexenyl hexanoate, n-hexyl-hexanoate, *β*-caryophyllene, and methyl dodecanoate. Six components were identified with a spicy flavor, including ethanol, 1-propanol, pentanoic acid, 5-methylfurfural, *α*-phellandrene, and 2-pentadecanone, which aligns with the apple-like fragrance of chamomile [2,30].

The aroma components identified in this experiment, including *β*-caryophyllene, *α*-pinene, *β*-pinene, and *α*-phellandrene, are consistent with those reported in the literature for chamomile [31], further enhancing the credibility of the electronic nose in characterizing odors. The relative abundance of compounds such as butanoic acid, ethyl ester, 3-nonanone, and geosmin was highest among the odor components and was classified as fruity and spicy odors, likely representing key factors influencing the aroma of chamomile. Since high temperatures can lead to the degradation of volatile components in chamomile, the electronic nose, to a certain extent, accurately reflects the information on volatile components of chamomile, providing a reference for the analysis of chamomile’s odor components [32,33].

#### 3.4.2. PCA

**Figure 5:**
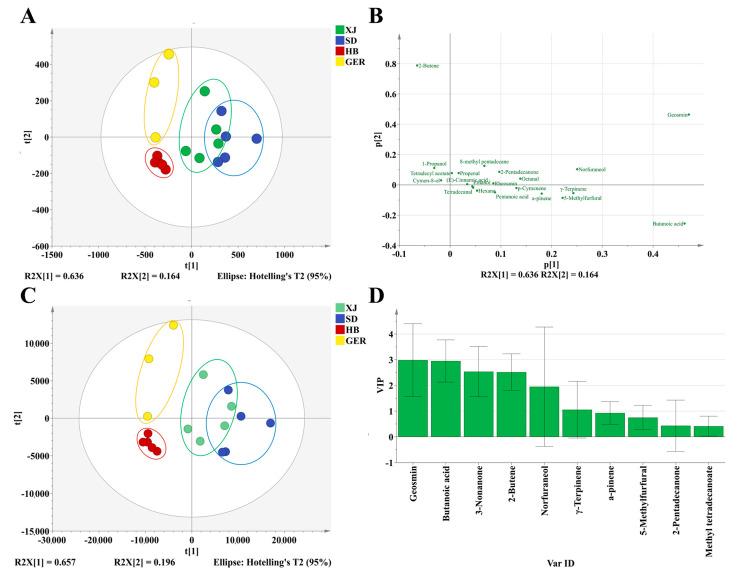
(**A**) Score plots of PCA model; (**B**) Loading scatter plots of PCA model; (**C**) Score plots of PLS–DA model; (**D**) Variable importance factor (VIP) plots.

The ultra-fast gas chromatography electronic nose can detect distinctive aromas quickly, making it suitable for the rapid identification of the areas of medicinal materials. Based on the aroma information obtained from the ultra-fast gas chromatography electronic nose, the common odor peak areas of chamomile (with an integrated peak area greater than 1000) were used as variables for PCA analysis using SIMCA 14.1 software, as shown in Figure 5. The results show that the two principal components from the PCA score plot account for a cumulative contribution rate of 89.60% (PC1 63.60%, PC2 16.40%), with a model predictive Q2 value of 0.896 (>0.5), indicating that the model effectively reflects the odor information present in the samples.

There is clear separation between the samples from different areas, suggesting that the aroma components may be area-specific. PCA results show that XJ and SD samples can be distinguished; however, they are closer in distance compared to the HB and GER samples. This indicates that while geographical location and climate differences between XJ and SD contribute to their odor differences, they do not fully account for them. This suggests that other factors, such as germplasm or cultivation methods, may influence the aroma of chamomile. It also indicates that while they have distinct profiles, there might be some similarities in certain components. The inter-group variation among Shandong samples is larger, which may be related to coastal climate conditions. High temperatures and heavy rainfall could affect the harvesting and drying of chamomile, ultimately impacting quality.

The figure illustrates that samples from Hebei showed smaller inter-group differences, which may be related to geographical location, agricultural production methods, similar climatic conditions, and farming practices. These results indicate that the ultra-fast gas chromatography electronic nose can effectively analyze the aroma components of chamomile. The detected aroma characteristics have the potential to serve as discriminative indicators for identifying different chamomile samples.

#### 3.4.3. Establishment of the DFA Discrimination Model

Discriminant factor analysis (DFA) is a model that extends differentiation among groups while compressing variations within the same group based on PCA [34]. This method facilitates a more intuitive discrimination of odor differences in chamomile from various sources and validates the PCA results.

**Figure 6:**
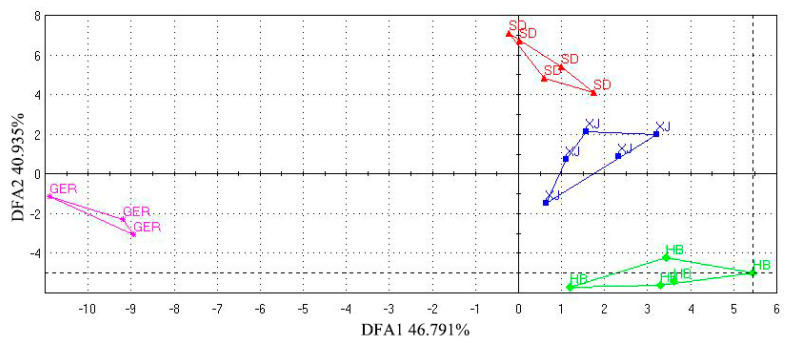
Discriminant function analysis.

In this study, DFA analysis of odor data from different chamomile samples was performed by Alpha Soft 17.0 software, which is a unique software package included with the electronic nose (Figure 6). The results show that the two-dimensional DFA score plot distinctly categorizes chamomile samples from different origins into four areas. The discriminant factors DF1 and DF2 contribute 46.791% and 40.935% respectively, with a cumulative contribution of 87.726%. This indicates that the DFA model effectively differentiates the geographical areas of chamomile and further verifies that the ultra-fast gas chromatography electronic nose can be used for rapid and accurate identification of chamomile from different sources [35]. The two-dimensional DFA plot clearly divides chamomile from different sources into four distinct areas, with the largest model distance between the samples from Germany and others, indicating significant odor differences between chamomile from China and Germany. The odor characteristics of chamomile are influenced by various factors such as geographical location, climatic conditions, and production methods.

#### 3.4.4. Study on Odor Biomarkers of Chamomile from Different Areas

PCA cannot overlook intra-group differences and random errors when identifying differential components. A PLS–DA model was employed to further explore the odor differences of chamomile samples (Figure 5). The results divided 18 batches of chamomile into four categories, with samples from the same area clustering together. The model’s R2X = 0.996, R2Y = 0.727, and Q2 = 0.509, indicate good explanatory and predictive capabilities. A 200 permutation test yielded R2X = 0.293 and Q2Y = −0.416, which are both below 0.3 and 0.05, respectively, demonstrating the model’s reliability and the absence of overfitting (Figure S1).

The VIP scores were plotted (Figure 5) to identify the characteristic aroma components affecting classification. Components with VIP values greater than 1 were selected as key aroma indicators influencing different chamomile samples. The analysis identified six components with VIP values greater than 1: geosmin, butanoic acid, 3-noanone, 2-butene, norfuraneol, and γ-terpinene. These components serve as important variables in the model analysis and may represent the substances responsible for the aroma differences among chamomile samples. The aroma characteristics identified by the electronic nose suggest that these six components predominantly exhibit fruity and spicy odors.

**Figure 7:**
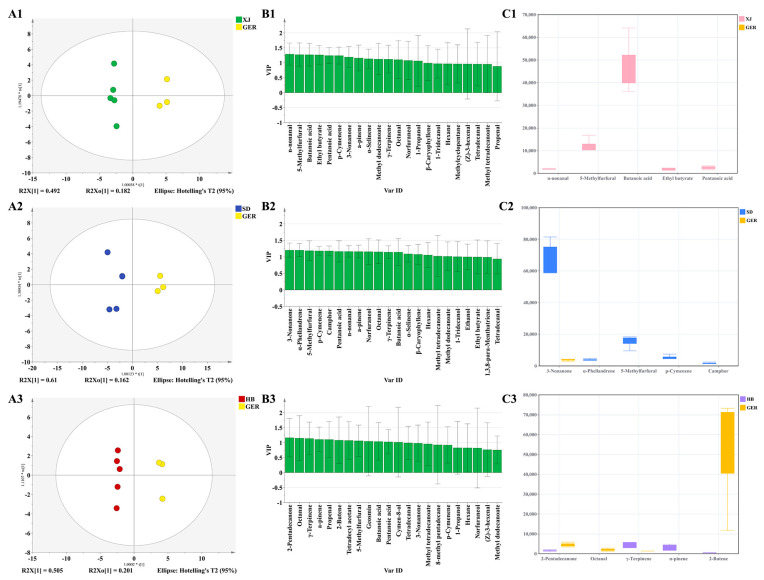
Score scatter plots (**A1**–**A3**); VIP plots (**B1**–**B3**); Comparison of the main difference compounds (**C1**–**C3**).

PCA and PLS–DA score plots showed that the GER samples were considerably distant from the CN samples, indicating a completely diverse aroma profile. Therefore, an OPLS–DA model was used to further explain the flavor differentials between GER and CN samples, and the scatter plots and flavor variables (VIP > 1.0) are shown in Figure 7. Comparing GER to XJ samples, n-nonanal, 5-methyfurfural, butanoic acid, and ethyl butyrate had a greater effect in discrimination, exhibiting the main flavors of spicy and fruity. The main flavor differentials between GER and SD samples were 3-nonanone, *α*-phellandrene, 5-methyfurfural and *p*-cymenene, also exhibiting spicy and fruity flavors. GER samples showed differences from HB samples in flavor compounds such as 2-pentadecanone, octanal, *γ*-terpinene, and *α*-pinene. Overall, the primary odor components distinguishing GER from CN samples were characterized by spicy and fruity notes.

Generally, chamomile is known for its apple-like fragrance and is extensively used in various fragrances and flavorings. Although highly responsive irritant components can be characterized by the electronic nose system, the influence of other odors, such as minty or woody, should not be underestimated. Only by integrating all aspects of the odor profiles can the authenticity of the quality assessment for chamomile be ensured.

## 4. Discussion

Previous studies have focused on the volatile oil components of chamomile and their effects. The characteristic components of the oil are mainly chamazulene and oxygenated sesquiterpenes, including bisabolol oxide B, α-bisabolol, bisabolone oxide, and bisabolol oxide A [18]. Our research confirmed the existence of these components in different chamomile samples, suggesting that these compounds can serve as identification markers for chamomile. Additionally, differences in the volatile oil content of chamomile were discovered; the SD samples exhibited the highest concentrations of chamazulene and bisabolol, indicating superior quality [24]. PCA analysis based on GC–MS indicated that volatile oil components could be used to distinguish chamomile samples. Furthermore, PLS–DA analysis initially explored the key volatile oil components distinguishing the different groups of chamomile, identifying (*E*)-*β*-famesene, chamazulene, *α*-bisabolol oxide B, spathulenol, and *α*-bisabolone oxide A.

The ultra-fast gas chromatography electronic nose technology can extract major aroma components from chamomile and provide corresponding sensory information within 110 s [36]. Sensory evaluation results indicate that the primary aromas of chamomile are concentrated on sweet and spicy odors, consistent with the general records of chamomile scent [2,30]. The electronic nose results indicate that the SD samples have the most intense odor, possess higher aromaticity, and are of better quality. This is the first attempt to distinguish different chamomile samples using the electronic nose, with the finding that the electronic nose results could preliminarily discriminate chamomile samples from four areas. Subsequently, DFA was used to perform group discrimination from different dimensions, validating the PCA results. Finally, PLS-DA was employed to identify potential differentiators between GER and CN samples: geosmin, butanoic acid, 3-nonanone, 2-butene, norfuraneol, and γ-terpinene. Additionally, we conducted OPLS–DA to analyze the differentiating compounds between GER and each specific CN sample. The combination of the two techniques not only provides the relative content of volatile oil components from chamomile originating in different areas but also analyzes the comprehensive aroma characteristics, offering a rapid, accurate, and feasible strategy for analyzing the volatile components of chamomile. This provides new insights for the quality evaluation of chamomile and technological support for the future development of chamomile tea and the traditional Chinese medicine industry.

Compared to traditional methods relying on human olfactory experience to judge the authenticity and quality of aromatic products, the ultra-fast gas chromatography electronic nose offers unique advantages of speed, non-destructiveness, objective evaluation results, and effective avoidance of human-related errors. This technology enables the swift identification of odor profiles and precise differentiation based on the region and authenticity of samples [37]. Although electronic nose technology is widely used in odor recognition and related fields, its application in aromatic product research may encounter challenges in specificity and exclusivity of identification results. This is primarily because electronic nose technology often targets specific natural substances, such as hydrocarbons and alcohols, when developing odor component databases.

The multivariate analysis results showed some distinction between different groups. However, the representativeness of these results is limited by the small number of chamomile samples analyzed in this study. Future research will increase the sample size to better capture the differences between various types of chamomile.

## 5. Conclusions

This study is the first to explore the application of GC–MS and ultra-fast gas chromatography electronic nose technology for distinguishing the components of chamomile samples. A comprehensive comparison of the volatile components of chamomile at different boiling points was conducted, and a rapid analysis technique based on the ultra-fast gas chromatography electronic nose was developed specifically for chamomile. Using GC–MS and the ultra-fast gas chromatography electronic nose, 16 volatile oil components and forty aroma components were identified. This study provided a comprehensive analysis of the volatile oils and aromatic components of chamomile, examining both the identified volatile oil components and those volatiles present at low temperatures. The analysis of high-temperature (GC–MS) and low-temperature (e-nose) products revealed that this chamomile contains characteristic components, such as bisabolol derivatives and chamazulene. The aroma profile of chamomile was attributed to fruity and spicy notes, aligning with the “apple-like” meaning of its name. This analysis offers viable strategies for quality assessment and differentiation of chamomile from various sources, potentially reducing adulteration in high-value agricultural and herbal products and enhancing consumer satisfaction. The findings are constrained by the limited sample size. Future research should include a larger number of samples to enhance these findings.

## Figures and Tables

**Table 1:** Sample information.

No.	Sources	No.	Sources	No.	Sources
S1	XJ	S7	SD	S13	HB
S2	XJ	S8	SD	S14	HB
S3	XJ	S9	SD	S15	HB
S4	XJ	S10	SD	S16	GER
S5	XJ	S11	HB	S17	GER
S6	SD	S12	HB	S18	GER

No.: The number of each sample; Sources: Source information of the sample; XJ: Xinjiang Province, China; SD: Shandong Province, China; HB: Hebei Province, China; GER: Samples collected in Germany.

## Data Availability

The original contributions presented in the study are included in the article/supplementary material, further inquiries can be directed to the corresponding authors.

## Supplementary Materials

The following are available online at https://www.mdpi.com/article/10.3390/foods13121865/s1, Table S1: The formula, and sensory description of flavor components in chamomile by fast GC e-nose. Table S2: Volatile oil composition of chamomile. Table S3: Electronic nose raw data. Figure S1: Results of Permutation. Figure S2: Results of Single-Factor Investigations.
