# Supplementary material for: GC–MS Combined with Fast GC E-Nose for the Analysis of Volatile Components of Chamomile (Matricaria chamomilla L.)
Source: Foods. 2024 Jun 13;13(12):1865. doi: 10.3390/foods13121865 (PMC11203138; doi:10.3390/foods13121865)
Supplement: Supplementary file 1 [file foods-13-01865-s001.zip › foods-3022205-supplementary.pdf]

foods

MDPI

GC–MS Combined with Fast GC E-Nose for the Analysis of Volatile Components of Chamomile (*Matricaria chamomilla* L.)

Jiayu Lu [1,2], Zheng Jiang [1,2], Jingjie Dang [1,2], Dishuai Li [1,2], Daixin Yu [1,2], Cheng Qu [3,*] and Qinan Wu [1,2,*]

[1] Jiangsu Collaborative Innovation Center of Chinese Medicinal Resources Industrialization, Nanijing University of Chinese Medicine, Nanjing 210023, China; lujiayu5210@163.com (J.L.); jyjiangzheng@163.com (Z.J.); 20223110@njucm.edu.cn (J.D.); 20210651@njucm.edu.cn (D.L.); yudaixin0616@163.com (D.Y.)
[2] State Key Laboratory on Technologies for Chinese Medicine Pharmaceutical Process Control and Intelligent Manufacture, Nanjing University of Chinese Medicine, Nanjing 210023, China
[3] School of Pharmacy, Nanjing University of Chinese Medicine, Nanjing 210023, China
* Correspondence: qucheng@njucm.edu.cn (C.Q.); wuqn@njucm.edu.cn (Q.W.)

Table S1. The formula, and sensory description of flavor components in chamomile by fast GC e-nose.

MXT-5		MXT-1701		CAS	Formula	Nmae	Score	Sensory descriptors
RT	RI	RT	RI					
15.94	418	14.50	576	107-01-7	C_4H_8	2-Butene	86.81	Aromatic
16.96	439	14.50	576	107-02-8	C_3H_4O	Propenal	75.19	Almond; Cherry
18.16	463	14.54	577	64-17-5	C_2H_6O	Ethanol	95.25	Spicy; Ethanol
22.64	552	18.12	694	71-23-8	C_3H_8O	1-Propanol	86.95	Spicy; Ethanol
26.10	621	15.18	598	110-54-3	C_6H_{14}	Hexane	90.58	Gasoline; Alkane
26.98	639	16.34	636	96-37-7	C_6H_{12}	methylcyclopentane	81.25	Gasoline; Gasoline
37.08	761	25.72	834	623-43-8	$C_5H_8O_2$	Methyl crotonate	95.03	Fruity; Green
39.32	781	27.16	853	105-54-4	$C_6H_{12}O_2$	Ethyl butyrate	83.57	Fruity; Sweety
41.90	804	30.26	895	6789-80-6	$C_6H_{10}O$	(Z)-3-hexenal	87.58	Fruity; Oak
47.04	855	32.28	919	7452-79-1	$C_7H_{14}O_2$	Butanoic acid	96.01	Fruity; Apple
52.52	911	44.24	1066	109-52-4	$C_5H_{10}O_2$	Pentanoic acid	72.63	Spicy; Sweety
54.16	932	38.06	985	7785-26-4	$C_{10}H_{16}$	*a*-pinene	83.14	Resin; Terpenoids
55.74	951	48.72	1130	620-02-0	$C_6H_6O_2$	5-Methylfurfural	83.93	Spicy; Almond
57.04	967	39.82	1006	127-91-3	$C_{10}H_{16}$	*β*-Pinene	95.16	Resin; Wooden
58.69	987	33.47	932	123-35-3	$C_{10}H_{16}$	Myrcene	91.60	Fruity; Lemon
59.16	993	47.16	1106	124-13-0	$C_8H_{16}O$	Octanal	88.77	Fruity; Orange
59.74	1000	42.00	1036	99-83-2	$C_{10}H_{16}$	*a*-Phellandrene	79.98	Spicy; Orange
62.45	1041	56.66	1263	19322-27-1	$C_5H_6O_3$	norfuraneol	70.04	Sweety; Caramel
63.19	1055	47.70	1115	99-85-4	$C_{10}H_{16}$	*γ*-Terpinene	91.29	Fruity; Orange
64.42	1070	51.32	1170	925-78-0	$C_9H_{18}O$	3-Nonanone	93.95	Fruity; Vegetable

65.94	1093	50.30	1155	1195-32-0	$C_{10}H_{12}$	p-Cymenene	79.12	Fruity; Orange
66.64	1104	51.38	1171	124-19-6	$C_9H_{18}O$	n-nonanal	92.44	Fruity; Orange
68.62	1138	59.76	1323	21195-59-5	$C_{10}H_{14}$	1,3,8 para-Menthatriene	84.39	Terpenoids; Sulfurous
69.40	1152	59.76	1323	76-22-2	$C_{10}H_{16}O$	Camphor	77.12	Camphor; Green
70.90	1178	60.98	1348	1197-01-9	$C_{10}H_{14}O$	Cymen-8-ol	85.77	Fruity; Coumarin
71.78	1194	61.92	1368	33512	$C_8H_{10}O_3$	2,6-dimethoxy-phenol	97.70	Wooden; Smoke
74.24	1241	61.92	1368	1197-07-5	$C_{10}H_{16}O$	trans-Carveol	93.64	Coriander; Green
80.46	1370	65.10	1438	31501-11-8	$C_{12}H_{22}O_2$	(Z)-3-Hexenyl hexanoate	91.47	Fruity; Green
82.50	1415	65.10	1438	6378-65-0	$C_{12}H_{24}O_2$	N-hexyl-hexanoate	91.74	Fruity; Apple
83.56	1440	75.96	1683	140-10-3	$C_9H_8O_2$	(E)-Cinnamic acid	88.00	Sweety; Vegetable
84.71	1468	69.05	1528	19700-21-1	$C_{12}H_{22}O$	Geosmin	86.13	Beet; Soil
85.68	1489	68.68	1519	87-44-5	$C_{17}H_{28}$	*β*-Caryophyllene	92.74	Fruity; Green
86.30	1504	68.68	1519	473-13-2	$C_{15}H_{24}$	*a*-Selinene	91.27	Amber; Orange
86.82	1517	72.92	1614	111-82-0	$C_{13}H_{26}O_2$	Methyl dodecanoate	96.13	Fruity; Fatty
87.38	1531	68.68	1519	22306-28-1	$C_{16}H_{34}$	8-methyl pentadecane	94.28	-
87.84	1543	66.66	1473	5471-51-2	$C_{10}H_{12}O_2$	Rheosmin	97.55	-
88.52	1560	77.70	1722	112-70-9	$C_{13}H_{28}O$	1-Tridecanol	87.18	-
91.54	1636	77.70	1722	124-25-4	$C_{14}H_{28}O$	Tetradecanal	95.62	Amber; Orange
94.36	1707	80.08	1775	2345-28-0	$C_{15}H_{30}O$	2-Pentadecanone	96.45	Spicy; Vegetable
95.80	1743	81.08	1797	124-10-7	$C_{15}H_{30}O_2$	Methyl tetradecanoate	83.77	Coconut; Fatty
98.78	1818	84.32	1870	638-59-5	$C_{16}H_{32}O_2$	Tetradecyl acetate	91.46	Fatty; Waxy

[1] RT, MXT-5 and RT, MXT-1701: Retention time measured in column MXT-5 or MXT-1701; RI, MXT-5 and RI, MXT-1701: Retention index measured by n-alkanes in column MXT-5 or MXT-1701;CAS: Chemical Abstracts Service registry number.

Table S2. Volatile oil composition of chamomile.

Compound	RT	RI-m	RI-r	CAS	S1	S2	S3	S4	S5	S6	S7	S8	S9	S10	S11	S12	S13	S14	S15	S16	S17	S18
(*E*)-beta-farnesene	22.05	1459	1448	18794-84-8	1.02	1.14	1.47	0.63	1.28	1.06	2.00	1.94	0.77	1.27	0.08	0.07	0.07	0.00	0.00	0.03	0.06	0.13
Dehydrosesquicineole	22.33	1471	1473	211237-38-6	0.00	0.00	0.00	0.00	0.00	0.00	0.00	0.00	0.00	0.00	0.02	0.00	0.00	0.00	0.00	0.00	0.00	0.00
Germacrene D	22.68	1485	1477	23986-74-5	0.04	0.10	0.09	0.06	0.11	0.07	0.36	0.38	0.15	0.18	0.00	0.00	0.00	0.00	0.00	0.00	0.00	0.00
Bicyclogermacrene	23.06	1500	1492	24703-35-3	0.00	0.10	0.00	0.00	0.12	0.07	0.38	0.41	0.16	0.18	0.00	0.00	0.00	0.00	0.00	0.00	0.00	0.00

Spathulenol	25.21	1592	1568	6750-60-3	0.00	0.48	0.15	0.27	0.23	0.14	0.19	0.18	0.25	0.23	0.55	0.49	0.59	0.36	0.68	0.08	0.06	0.06
γ-Eudesmol	25.90	1622	1630	1209-71-8	0.00	0.10	0.00	0.10	0.09	0.09	0.10	0.12	0.08	0.08	0.10	0.07	0.09	0.08	0.15	0.05	0.03	0.04
β-Bisabolol	26.29	1640	1758	15352-77-9	0.00	0.05	0.00	0.00	0.00	0.05	0.00	0.05	0.00	0.00	0.03	0.04	0.04	0.00	0.06	0.00	0.00	0.00
γ-Cadinol	26.49	1649	1639	5937-11-1	0.00	0.05	0.06	0.00	0.00	0.00	0.03	0.03	0.00	0.00	0.00	0.00	0.00	0.00	0.00	0.00	0.00	0.00
α-Bisabolol oxide B	26.82	1663	1644	26184-88-3	0.67	2.92	0.70	2.34	2.82	2.62	2.30	2.62	3.36	2.63	1.51	1.40	1.44	0.88	2.04	1.17	1.26	0.83
Bisabolol oxide B	26.86	1665	1666	55399-12-7	0.00	0.21	0.14	0.15	0.00	0.00	0.11	0.00	0.00	0.00	0.00	0.00	0.00	0.00	0.00	0.06	0.04	0.06
α-Bisabolone oxide A	27.37	1688	1670	22567-38-0	0.39	1.31	0.60	1.41	0.98	1.04	1.47	1.31	2.14	1.76	1.21	1.07	1.22	0.72	1.38	0.45	0.54	0.31
α-Bisabolol	27.53	1695	1684	515-69-5	0.01	0.00	0.00	0.00	0.01	0.01	0.00	0.00	0.00	0.00	0.01	0.01	0.01	0.00	0.01	0.00	0.00	0.00
Chamazulene	28.42	1737	1707	529-05-5	0.09	1.08	0.17	1.28	0.93	0.57	1.92	2.05	3.79	2.37	0.52	0.51	0.57	0.17	0.47	0.18	0.31	0.17
α-Bisabolol oxide A	28.90	1759	1758	22567-36-8	0.61	0.31	0.85	0.32	0.23	0.25	0.39	0.40	0.44	0.42	0.25	0.26	0.21	0.13	0.25	0.25	0.32	0.21
(Z)-ene-yne-Dicycloether	31.00	1855	1849	4575-53-5	0.07	0.08	0.08	0.06	0.12	0.06	0.07	0.08	0.16	0.09	0.07	0.07	0.05	0.02	0.08	0.00	0.00	0.00
(Z)-18-Octadec-9-enolide	36.47	2157	2158	74992-69-1	0.04	0.04	0.00	0.00	0.03	0.00	0.00	0.00	0.00	0.00	0.05	0.05	0.07	0.05	0.06	0.12	0.15	0.10
Heptacosane	42.29	2502	2500	593-49-7	0.07	0.07	0.08	0.07	0.07	0.07	0.08	0.07	0.10	0.05	0.14	0.14	0.20	0.16	0.14	0.10	0.03	0.06
Essential oil (%)					0.75%	0.90%	1.00%	0.77%	0.92%	0.91%	1.20%	1.10%	0.86%	1.00%	0.63%	0.55%	0.52%	0.44%	0.43%	0.66%	1.05%	0.93%

[2] RT: relative retention time in GC-MS; RI-m: the actual retention index calculated by n-alkanes; RI-r: the theoretical retention index in the NIST 14 library; CAS: Chemical Abstracts Service registry number; S1~S18: the content of each sample compared with the internal standard(μg/mL).

Table S3. Electronic nose raw data.

ID	S1	S2	S3	S4	S5	S6	S7	S8	S9
2-Butene	759.73	65103.63	1926.23	913.93	1101.27	1098.17	17751.00	1334.97	22260.40
Propenal	0.00	1874.43	0.00	2095.53	1734.67	2144.97	3113.40	1542.33	2181.33
Ethanol	0.00	0.00	0.00	0.00	0.00	0.00	1331.93	1345.47	1215.23
1-Propanol	0.00	1812.70	0.00	0.00	0.00	1086.30	0.00	0.00	1039.83
Hexane	1413.77	1442.03	0.00	1842.60	1701.47	2573.47	1375.23	2227.33	1316.00
Methylcyclopentane	1101.57	1025.63	0.00	1141.00	1544.90	1630.97	0.00	1506.77	0.00
Ethylbutyrate	1288.90	1442.63	2349.67	1655.70	2217.40	2232.27	0.00	1999.20	2163.43
(Z)-3-hexenal	0.00	0.00	0.00	0.00	0.00	0.00	0.00	0.00	0.00
Butanoicacid	39825.90	36090.40	64121.77	47016.93	52171.73	63605.33	30753.70	85530.10	87087.07
Pentanoicacid	1541.80	2946.93	2015.40	1996.03	3305.83	3454.83	1886.03	3532.13	3321.63
α-pinene	3964.10	12011.33	5066.50	9059.00	11462.07	6641.10	12468.67	16600.90	12740.53
5-Methylfurfural	10386.57	10242.27	16823.20	10652.50	12929.70	14065.03	9431.53	18276.87	17622.17

β-Pinene	2825.27	0.00	3759.80	0.00	0.00	0.00	0.00	0.00	0.00
Myrcene	0.00	0.00	0.00	0.00	0.00	1411.57	0.00	0.00	0.00
Octanal	5071.87	5695.73	8308.40	3830.50	4472.23	4511.77	5300.87	8600.37	6826.03
α-Phellandrene	0.00	4068.90	0.00	2417.53	2901.03	3362.17	3352.40	4638.93	3901.97
Norfuraneol	13087.60	29475.40	26534.43	12418.77	15652.10	19603.53	14096.07	23196.83	22225.17
γ-Terpinene	3934.27	15148.43	6726.33	12691.27	19262.80	11272.73	22346.10	30543.47	21975.97
3-Nonanone	19686.63	53662.37	31473.97	44372.27	54650.00	58637.87	58901.03	81433.23	75145.13
p-Cymenene	1686.13	4190.17	2799.60	3223.77	4061.13	4378.00	5590.67	7329.37	5053.70
n-nonanal	1560.13	1899.97	2609.00	1967.13	1848.50	2787.47	2879.03	4546.57	2688.83
1,3,8para-Menthatriene	0.00	0.00	1088.83	0.00	1237.03	0.00	2007.83	2998.63	1786.87
camphor	0.00	0.00	0.00	974.83	0.00	1373.33	1576.70	2549.07	1742.67
Cymen-8-ol	1108.40	0.00	1104.30	0.00	0.00	0.00	0.00	0.00	0.00
2,6-dimethoxy-phenol	1438.27	1109.00	1981.37	0.00	0.00	0.00	0.00	0.00	0.00
trans-Carveol	0.00	0.00	1761.77	0.00	0.00	0.00	0.00	0.00	0.00
(Z)-3-Hexenylhexanoate	0.00	0.00	0.00	0.00	1431.77	1001.40	2276.87	3302.57	0.00
N-hexyl-hexanoate	0.00	0.00	0.00	0.00	1064.13	885.43	0.00	1270.37	0.00
(E)-Cinnamicacid	0.00	0.00	1596.70	0.00	0.00	1066.33	0.00	1034.03	0.00
Geosmin	55573.73	60881.90	112647.80	53439.87	89852.30	112605.77	114816.70	129848.17	40686.50
β-Caryophyllene	0.00	3165.20	1211.83	3023.50	2076.07	3725.37	4616.73	5347.63	1834.57
α-Selinene	0.00	1076.03	1023.40	920.90	1422.33	1468.30	1794.07	2091.00	976.57
Methyldodecanoate	1141.80	1871.83	1953.47	2264.87	2651.07	3926.50	3601.00	5437.80	1375.33
8-methylpentadecane	0.00	1386.10	3178.50	1246.83	1850.03	2136.53	3247.63	4391.73	1331.03
Rheosmin	1580.07	1732.53	0.00	1565.50	2461.57	2460.23	3832.57	5646.20	1310.87
1-Tridecanol	0.00	1235.83	1502.37	1177.00	1655.07	1926.73	2619.20	3772.20	1025.37
Tetradecanal	0.00	1214.87	1275.77	0.00	2401.50	1869.07	2514.83	2116.43	1598.35
2-Pentadecanone	2512.53	2492.53	3742.90	1322.43	9398.00	5122.23	8470.77	7775.10	8841.70
Methyltetradecanoate	2041.27	2530.77	2690.80	2151.07	4356.57	4535.70	7265.13	5108.70	6779.10
Tetradecylacetate	2114.10	0.00	3467.87	0.00	1709.83	0.00	2113.90	0.00	0.00

ID	S10	S11	S12	S13	S14	S15	S16	S17	S18
2-Butene	15453.90	413.00	326.94	541.57	437.23	361.97	69229.50	73239.37	11689.13
Propenal	1687.17	1656.57	0.00	0.00	0.00	1196.13	2680.07	1976.20	3484.53
Ethanol	1307.40	0.00	0.00	0.00	0.00	0.00	0.00	0.00	0.00
1-Propanol	1260.07	0.00	0.00	1079.27	0.00	2470.63	2685.50	3379.37	1027.57
Hexane	1062.37	1305.40	1275.99	0.00	0.00	1103.63	0.00	0.00	0.00
Methylcyclopentane	0.00	1241.27	0.00	0.00	0.00	0.00	0.00	0.00	0.00
Ethylbutyrate	2113.10	0.00	0.00	0.00	1057.57	0.00	0.00	0.00	0.00

(Z)-3-hexenal	0.00	1720.70	0.00	0.00	0.00	2890.20	3723.83	0.00	2307.57
Butanoicacid	91001.33	7112.23	3888.48	5250.17	20890.07	13121.03	0.00	0.00	0.00
Pentanoicacid	2813.30	1172.67	0.00	1295.67	1381.90	1494.97	0.00	0.00	0.00
α-pinene	10498.73	3970.93	1658.28	1774.40	4717.53	4330.73	0.00	0.00	0.00
5-Methylfurfural	16116.10	1809.30	840.39	1142.87	4081.90	3074.50	0.00	0.00	0.00
β-Pinene	0.00	0.00	0.00	0.00	0.00	0.00	0.00	0.00	0.00
Myrcene	0.00	0.00	0.00	0.00	0.00	0.00	0.00	0.00	0.00
Octanal	5815.93	0.00	0.00	0.00	0.00	0.00	1254.50	1946.27	2810.00
α-Phellandrene	3433.43	0.00	0.00	0.00	0.00	0.00	0.00	0.00	0.00
Norfuraneol	18564.70	1271.47	553.15	623.50	1925.73	1635.27	3386.67	6801.70	0.00
γ-Terpinene	17006.97	4203.13	2975.24	2987.33	5673.67	5740.60	0.00	1350.00	0.00
3-Nonanone	60307.17	11423.87	5198.19	6392.27	21668.47	18359.43	4088.63	4111.10	2843.93
p-Cymenene	4424.33	901.27	0.00	0.00	1764.83	1468.80	0.00	0.00	0.00
n-nonanal	2093.33	0.00	0.00	0.00	0.00	0.00	0.00	0.00	0.00
1,3,8para-Menthatriene	1481.30	0.00	0.00	0.00	0.00	0.00	0.00	0.00	0.00
camphor	1343.37	0.00	0.00	0.00	0.00	0.00	0.00	0.00	0.00
Cymen-8-ol	0.00	0.00	0.00	0.00	0.00	0.00	1399.50	0.00	1290.00
2,6-dimethoxy-phenol	0.00	0.00	0.00	0.00	0.00	0.00	0.00	0.00	0.00
trans-Carveol	0.00	0.00	0.00	0.00	0.00	0.00	0.00	0.00	0.00
(Z)-3-Hexenylhexanoate	0.00	0.00	0.00	0.00	0.00	0.00	0.00	0.00	0.00
N-hexyl-hexanoate	0.00	0.00	0.00	0.00	0.00	0.00	0.00	0.00	0.00
(E)-Cinnamicacid	0.00	0.00	0.00	0.00	0.00	0.00	0.00	0.00	0.00
Geosmin	44809.70	10433.40	19981.88	6580.30	8910.20	8476.73	30665.90	82372.40	25840.80
β-Caryophyllene	2881.97	0.00	0.00	0.00	0.00	0.00	0.00	0.00	0.00
α-Selinene	763.30	0.00	0.00	0.00	0.00	0.00	0.00	0.00	0.00
Methyldodecanoate	1754.87	0.00	1237.49	0.00	0.00	0.00	0.00	1416.80	0.00
8-methylpentadecane	1399.57	0.00	1268.35	0.00	0.00	0.00	1463.33	4185.30	0.00
Rheosmin	1362.10	0.00	0.00	0.00	0.00	0.00	0.00	0.00	2156.43
1-Tridecanol	1034.13	0.00	0.00	0.00	0.00	0.00	0.00	1094.47	0.00
Tetradecanal	0.00	0.00	1525.54	1368.37	870.03	1571.03	0.00	0.00	0.00
2-Pentadecanone	2631.07	1457.70	1502.56	1718.83	1054.37	2089.23	3408.67	5812.40	4252.50
Methyltetradecanoate	3956.00	2571.93	3466.89	3800.03	1721.23	3065.97	1468.40	2271.27	1852.00
Tetradecylacetate	0.00	0.00	1103.28	0.00	0.00	0.00	1027.77	2104.77	1017.13

[3] ID: S1-S18 represent different chamomile samples; the leftmost column lists the name of identified compounds for each peak; the data represent the signal peak areas analysed by the Rapid Gas Chromatography Electronic Nose.

Figure S1. Results of Permutation.

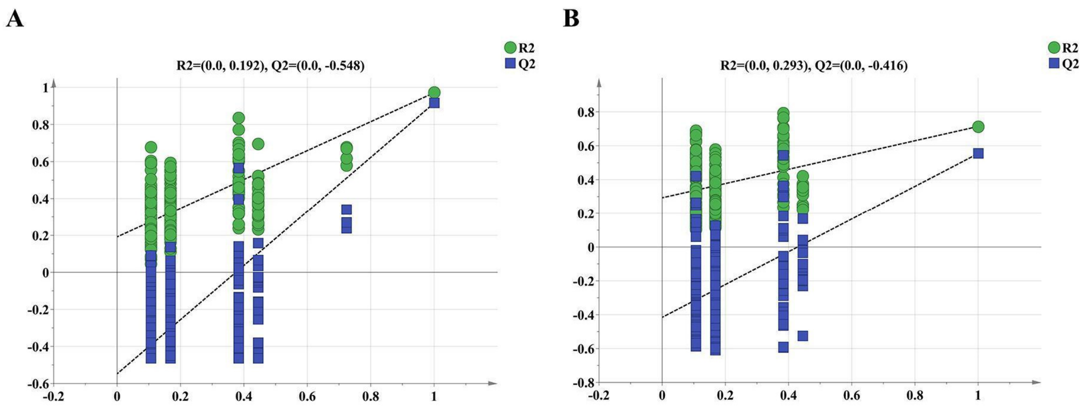

[4] A: PLSDA Permutation result of GC-MS; B: PLSDA Permutation result of electronic nose.

Figure S2. Results of Single-Factor Investigations

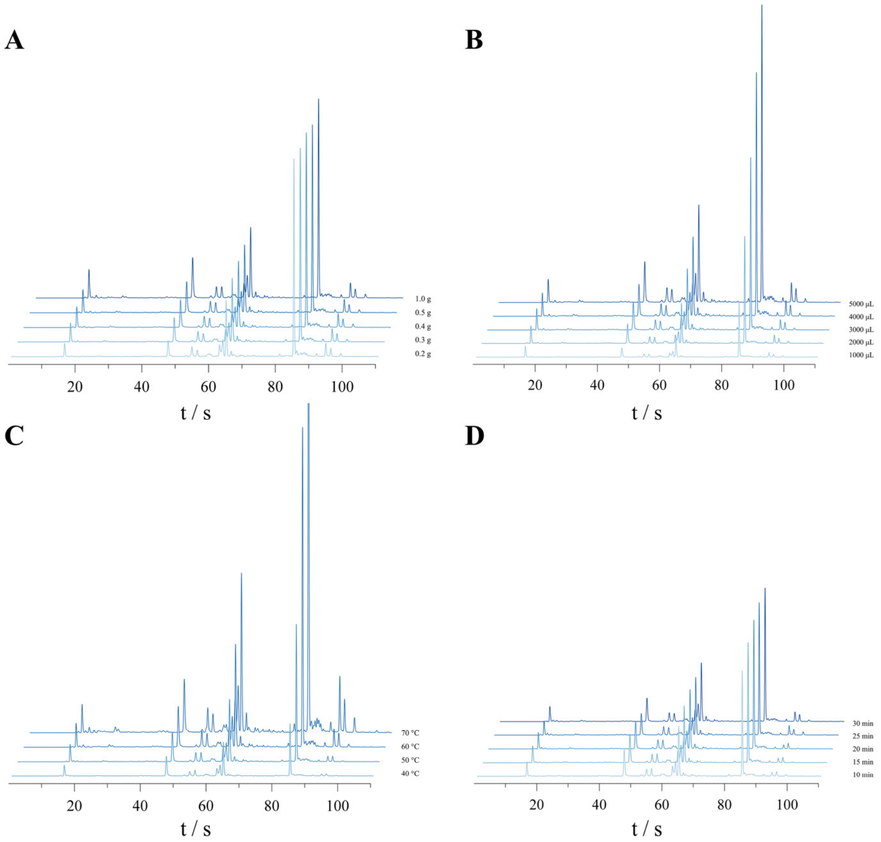

[5] A: Chromatograms of Sample Quantity; B: Chromatograms of Injection Volume; C: Chromatograms of Incubation Temperature; D: Chromatograms of Incubation Time.
